# Zebrafish fin regeneration after cryoinjury-induced tissue damage

**DOI:** 10.1242/bio.016865

**Published:** 2016-05-23

**Authors:** Bérénice Chassot, David Pury, Anna Jaźwińska

**Affiliations:** Department of Biology, University of Fribourg, Chemin du Musée 10, Fribourg 1700, Switzerland

**Keywords:** Injury model, Caudal fin, Cryolesion, Histolysis, Limb regeneration, Appendage

## Abstract

Although fin regeneration following an amputation procedure has been well characterized, little is known about the impact of prolonged tissue damage on the execution of the regenerative programme in the zebrafish appendages. To induce histolytic processes in the caudal fin, we developed a new cryolesion model that combines the detrimental effects of freezing/thawing and ischemia. In contrast to the common transection model, the damaged part of the fin was spontaneously shed within two days after cryoinjury. The remaining stump contained a distorted margin with a mixture of dead material and healthy cells that concomitantly induced two opposing processes of tissue debris degradation and cellular proliferation, respectively. Between two and seven days after cryoinjury, this reparative/proliferative phase was morphologically featured by displaced fragments of broken bones. A blastemal marker *msxB* was induced in the intact mesenchyme below the damaged stump margin. Live imaging of epithelial and osteoblastic transgenic reporter lines revealed that the tissue-specific regenerative programmes were initiated after the clearance of damaged material. Despite histolytic perturbation during the first week after cryoinjury, the fin regeneration resumed and was completed without further alteration in comparison to the simple amputation model. This model reveals the powerful ability of the zebrafish to restore the original appendage architecture after the extended histolysis of the stump.

## Abstract (precis)

**Summary:** Fin cryolesion resulted in histolysis and a delayed tissue loss. Despite prolonged destruction of the stump architecture, fin regeneration resumed and was normally completed, revealing robustness of the regenerative capacity.

## INTRODUCTION

In mammals, such as mice, humans and other primates, the digit tips are the only part of the limbs that can regenerate after amputation (Shieh and Cheng, 2015; Simkin et al., 2015a). This capacity depends on the remaining nail structure and phalangeal bone, which play important roles as sources and coordinators of regenerative signals (Rinkevich et al., 2011; Takeo et al., 2013; Yu et al., 2012). The murine digit tip regeneration proceeds through a series of initial reparative events, namely blood clotting, inflammation and histolysis, followed by subsequent regenerative processes of epidermal closure, progenitor cell activation and redifferentiation (Lehoczky et al., 2011; Rinkevich et al., 2011; Simkin et al., 2015a,b; Wu et al., 2013). In contrast to the level-restricted regeneration in mammals, whereby the digits amputated proximally to the nail fail to regenerate, fish and urodeles possess the ability to restore the missing part of their appendages from any proximo-distal position of their extremities. This process involves wound healing, activation of the stump margin and blastema formation, which contains tissue-specific progenitor cells for the new outgrowth (Bryant and Gardiner, 2016; Godwin and Rosenthal, 2014; Knopf et al., 2011; Kragl et al., 2009; McCusker et al., 2015; Simon and Tanaka, 2013; Singh et al., 2012; Sousa et al., 2011; Stewart and Stankunas, 2012; Tu and Johnson, 2011). Although several cellular events underlying mammalian and non-mammalian appendage restoration are comparable, the endogenous regenerative competence and the origin of the inductive mechanisms seem to be different among these vertebrates.

The zebrafish represents a valuable model organism to study organ regeneration in vertebrates (Gemberling et al., 2013; Jaźwińska and Sallin, 2016). The caudal fin is a non-muscularized dermal fold that contains 16-18 main segmented and occasionally bifurcated rays spanned by soft inter-ray tissue (Pfefferli and Jaźwińska, 2015; Yoshinari and Kawakami, 2011). The length of each ray is stabilized by a pair of concave bones, called lepidotrichia, while the distal tip is supported by a brush-like bundle of fine spicules, named actinotrichia. These skeletal elements are located between the epidermis and the mesenchyme. Epimorphic regeneration of the fin is dependent on the epithelial-mesenchymal interactions that control the formation of a blastema, a pool of regeneration-competent cells from the stump (Blum and Begemann, 2012, 2015; Chablais and Jazwinska, 2010; Gemberling et al., 2013; Pfefferli and Jaźwińska, 2015; Wehner and Weidinger, 2015). The apical part of the blastema has been proposed to act as the upstream organizer of the regenerate through the Wnt signalling pathway, which regulates cell proliferation and plasticity indirectly via secondary signals, such as Fgf, Igf and Bmp (Wehner et al., 2014). A combination of various signalling pathways and epigenetic regulators are fundamental for the execution of the regenerative programme, which is completed at approximately 20 days post-amputation (dpa) (Blum and Begemann, 2012, 2015; Chablais and Jazwinska, 2010; Gemberling et al., 2013; Pfefferli and Jaźwińska, 2015; Tornini and Poss, 2014; Wehner and Weidinger, 2015).

Fin regeneration has mainly been studied after a simple removal of an organ part using an amputation procedure. Within a few hours after cutting the fin, the wound undergoes rapid re-epithelialization, as shown by live imaging of stump margin within the first few hours post-amputation (Jaźwińska et al., 2007). The inhibition of inflammation, either using a pre-treatment with high concentration of synthetic steroids or genetic ablation of macrophages, does not affect normal wound healing and blastema formation (Kyritsis et al., 2012; Petrie et al., 2014). A substantial inflammatory response and fibrosis have not been reported in the zebrafish fin, which typically occur after the loss of a mammalian limb. As opposed to the mammalian digits, the zebrafish fin stump initiates the regeneration programme immediately after the completion of wound healing, within the first 24 hours post-amputation (hpa). Thus, this model does not reproduce the mammalian limb injury response, which is typically associated with local tissue demolition and a burst of an inflammation at the cut site (Eming et al., 2014; Simkin et al., 2015a). It is noteworthy that amputation of the murine digit tip also triggers histolysis and the spontaneous release of a bone fragment before the transition to the rebuilding processes to replace the extremity (Fernando et al., 2011).

In this study, we aimed to mimic a reparative phase in the zebrafish fin by inducing conditions of extensive tissue damage without resection. Accordingly, we established a new wounding method based on cryoinjury. Here, we show that exposure to a frozen blade disrupts the tissue integrity resulting in a delayed loss of the dead portion of the fin between 24 and 48 hours post-cryoinjury (hpci). Importantly, the remaining stump comprised a histolytic zone with eroded bone segments and apoptotic cells. Despite the massive destruction of the stump architecture, regeneration resumed concomitantly to debris clearance at 3 to 5 days post-cryoinjury (dpci), and the outgrowth was completed as after amputation. This study demonstrates that the fin has a powerful ability to re-establish the regenerative programme despite the extensive disorganization of the stump tissues.

## RESULTS

### Cryoinjury of the caudal fin results in a delayed tissue loss

**Fig. 1.**
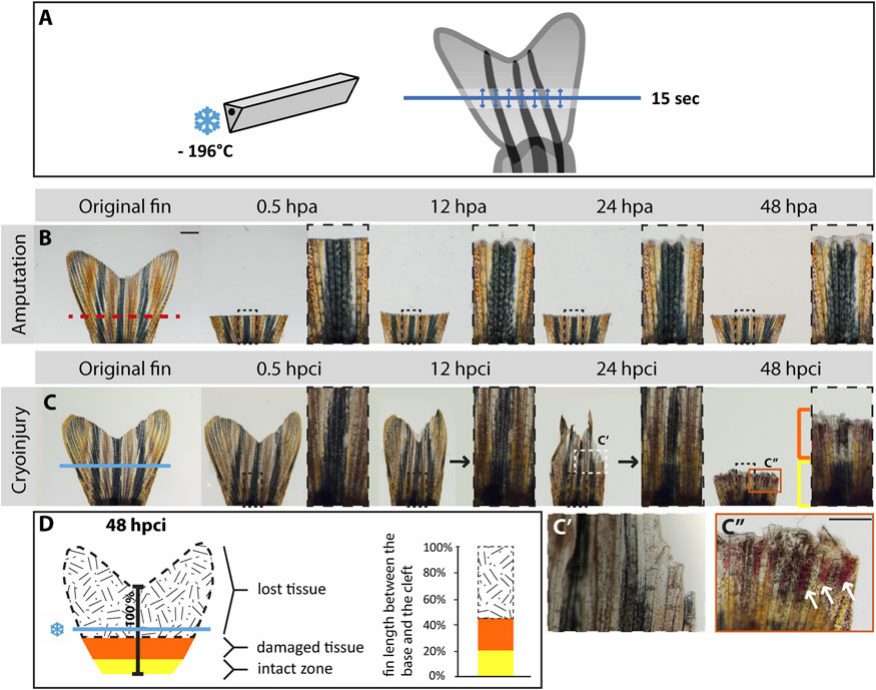
**Cryoinjury of the caudal fin results in spontaneous sloughing of destroyed tissue within two days after the damage induction.** (A) Schematic representation of the cryoinjury procedure. The cryotome blade (left side) was precooled in liquid nitrogen, and gently placed just above the fin (right side) for 15 s at the position demarcated by the blue line. The arrows indicate spreading of the cold from the blade in the adjacent tissue. (B,C) Time-lapse images of the caudal fin after amputation (B) and cryoinjury (C) showing the appendage before injury, and at 0.5 h, 12 h, 24 h and 48 h after the procedure; hpa, hours post-amputation; hpci, hours post-cryoinjury. As opposed to the transection model (B), in which a part of the fin is immediately removed from the amputation plane (red dashed line), the non-surgical exposure to the cold (C) along the cryoinjury plane (blue line) results in a progressive tissue detachment that is apparent between 12 and 48 hpci. At 48 hpa (B), the initiation of regeneration is detected by the presence of a whitish tissue containing the blastema and wound epidermis. At 48 hpci (C), the distal part of the stump contains a partially damaged tissue zone (orange bracket and frame) with affected pigmentation, blood clots and broken bones. The intact zone is restricted to the base of the fin (yellow bracket). Black arrows at 12 and 24 hpci indicate the plane with fading pigmentation. (C′) Magnified image of the white dashed line-framed area shown in above panel at 24 hpci. (C″) Magnification of the orange line-framed area shown in above panel at 48 hpci. White arrows indicate blood clots. (D) Schematic representation of the tissue damage after cryoinjury at 48 hpci, normalized to the distance between the base of the fin and the central cleft. The black-white patterned area corresponds to the sloughed part of the original fin. The remaining fin comprises partially damaged tissue (orange) and an intact stump (yellow). *N*=6. Scale bar in B=1 mm; C″=100 µm.

To investigate the impact of histolysis on the regenerative capacity of the zebrafish caudal fin, we developed a new method of cryoinjury. Specifically, a pre-cooled knife was placed for 15 s on the fin surface perpendicularly to the proximo-distal axis of the appendage at an equidistant position between the fin base and the central cleft (Fig. 1A). We observed that cold emanating from the metal knife led to the formation of ice crystals in the fin tissue within a distance of approximately 0.5 mm from the position of the tool, referred to as the cryoinjury plane (Fig. 1A). We expected that the process of freezing and thawing would destroy the cellular integrity. In the simple amputation model, the stump healed rapidly without restructuration of its original shape, and the regenerative programmes were activated within 48 hpa (Fig. 1B). At 48 hpa, a new tissue appeared above the amputation plane, which is known to contain wound epidermis and blastema. In contrast, the effects of cryoinjury were not morphologically detectable until 12 hpci, when the fin displayed mild distortion, such as a contracted shape and occasional indentations along the distal margin (Fig. 1C). Starting from this time point, a progressive detachment of tissue was observed, resulting in sloughing of the destroyed part at 48 hpci. To visualize the dynamics of morphological changes, we performed time-lapse imaging of the caudal fins within 2 days after cryolesion (Movie 1). A closer examination of the truncated fin at 48 hpci revealed an irregular margin with broken bones and dark necrotic patches (Fig. 1C″). Moreover, a region of approximately 1 mm underneath the stump margin contained abnormal pigmentation and excessive blood clots, indicating the presence of partially damaged tissue (Fig. 1C″). To quantify the extent of injury at 48 hpci, we performed morphometric analysis of the medial fin rays from the base to the central cleft. Using these measurements as a reference for the assessment of fin damage, we found that more than 50% of the medial ray length was completely shed off, 25% was partially distorted, and the remaining part of the stump appeared intact (Fig. 1D). Thus, cryoinjury had two consequences, namely a delayed sloughing of severely destroyed tissue distal to the cryoinjury plane, and a partial damage of the appendage architecture proximal to it.

**Fig. 2.**
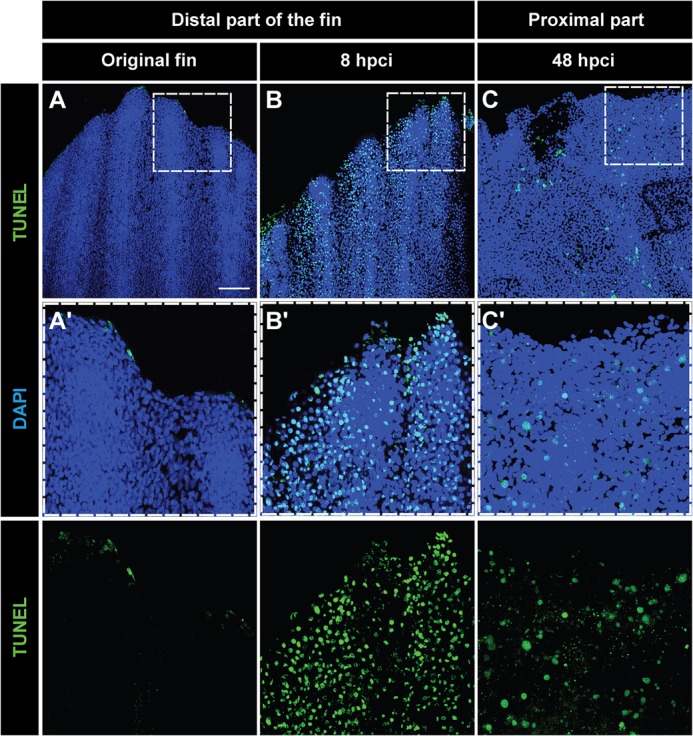
**Tissue loss after cryoinjury is associated with massive apoptosis.** (A-C) Whole-mount staining with DAPI (blue) and TUNEL (green) of the original fin (A), at 8 hpci (B) and at 48 hpci (C). (A′-C′) Magnifications of the framed areas of the upper images. (A,A′) Uninjured fins contain few apoptotic cells at the distal margin. (B,B′) Before sloughing of the cryoinjured fin part at 8 hpci, extensive apoptosis in the distal part of the extremity is observed. (C,C′) After truncation of the damaged fin part at 48 hpci, the margin of the remaining stump still contains apoptotic cells. *N*=4. Scale bar in A=100 µm.

Live imaging of the fins at 12 hpci revealed that the tissue loss was initiated at the distal end of the fin, which had not been covered with ice crystals during the freezing procedure. To understand the cellular causes underlying tissue rupture at this position, we analysed cell apoptosis using the terminal deoxynucleotidyl transferase digoxigenin-UTP nick end-labelling (TUNEL) assay. In uninjured control fins, only a few TUNEL-positive cells were detected in the appendage (Fig. 2A,A′). At 8 hpci, numerous apoptotic cells appeared in the distal portion of the fin (Fig. 2B,B′). We concluded that the entire portion of the fin above the cryoinjury plane has been severely affected by cryoinjury, which explains the complete detachment of this tissue within two days. After truncation of the apoptotic fin part at 48 hpci, TUNEL-positive cells were still detected in the remaining stump, indicating a partial destruction of the tissue (Fig. 2C,C′). The level of damage was, however, sufficient for maintaining the integrity of the distorted stump with the rest of the body.

To assess whether the massive apoptosis observed in the distal part of the appendage before fin sloughing was due to an interrupted blood flow at the level of the cryoinjury plane, we performed live-imaging of the *Tg(tie2:EGFP)* transgenic fish, in which GFP is expressed in endothelial cells (Fig. S1). In the fin, the veins and arteries are distributed parallel to the rays (Xu et al., 2014). We found that as soon as 10 min after cryoinjury, *tie2:EGFP* expression was nearly undetectable at the freezing plane, indicating damage to endothelial cells (Fig. S1A′,B′). Moreover, the injured tissue displayed no blood circulation (Movie 2). The part of the fin without blood flow corresponded to the region that eventually detached at 48 hpci. As expected, the proximal-most fin, referred as the intact zone, contained unaffected blood vessels (Fig. S1B′,C′,D′). We concluded that cryoinjury destroyed the vasculature at the site of freezing, resulting in an interrupted blood supply in the distal fin. Thus, the tissue distal to the cryoinjury was exposed to ischemia, which might directly cause massive apoptosis, leading to subsequent sloughing of the dead tissue.

**Fig. 3.**
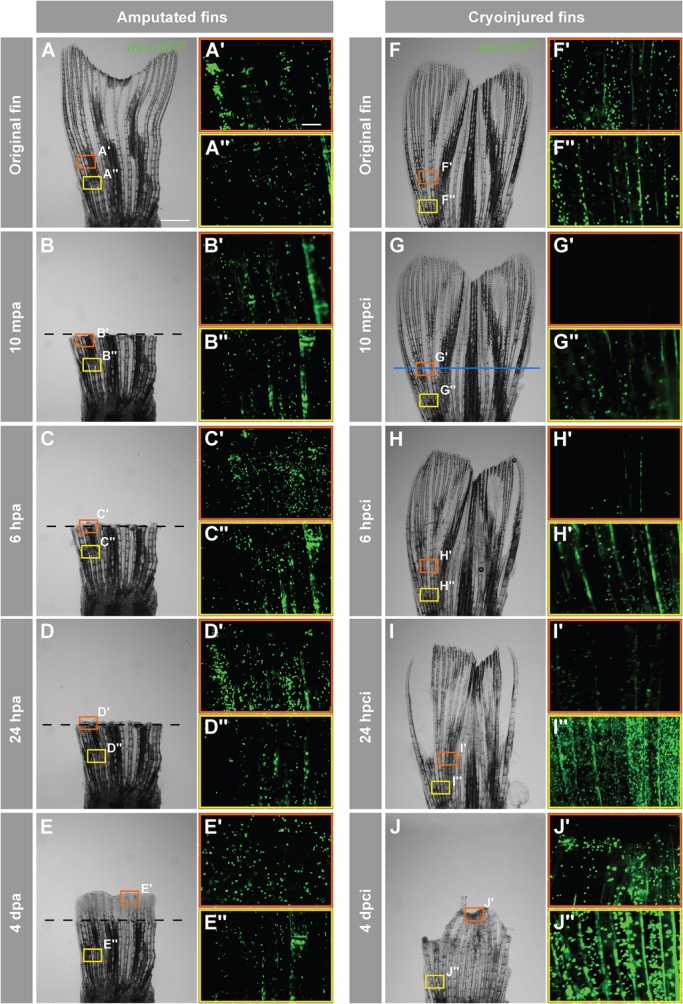
**Accumulation of neutrophils in the damaged tissue indicates an acute inflammatory response after cryoinjury.** *In-vivo* visualization of neutrophils in Tg(*mpx:GFP*) fish. (A-J) Time-lapse bright-field images of the same fins after amputation (A-E) and cryoinjury (F-J). Frames indicate the regions selected for fluorescence imaging of GFP-positive neutrophils, depicted in A′-Eʺ,F′-Jʺ. Middle region of the fin (orange box) at the level of the amputation plane (dashed line) and cryoinjury plane (blue line). The proximal part of the fin (yellow frame) that is remote from the injury site. In the amputation model (A-E), no change in the distribution of neutrophils is observed at either the amputation plane or proximal site (A′-Eʺ). After cryoinjury (F-J), neutrophils at the site of injury are destroyed (G′,H′), and they start to repopulate the stump margin at 24 hpci (I′) to reach normal distribution at 4 dpci (J″). The proximal intact stump comprises markedly increased numbers of neutrophils at 24 hpci (I″) and 4 dpci (J″). mpa, minutes post-amputation; mpci, minutes post-cryoinjury. *N*=4. Scale bar in A=1 mm, in A′=100 µm.

In the zebrafish, as in mammals, vasculature is the main path to distribute inflammatory cells to an injury site (Renshaw et al., 2006). A rapid recruitment of neutrophils represents the initial inflammatory response in the zebrafish larval fin (Li et al., 2012). To assess the distribution of neutrophils after fin cryoinjury, as compared to fin amputation, we used the Tg(*mpx:GFP*) zebrafish and performed time-lapse imaging. We focused on the area of injury and the intact part at the base of the appendage (Fig. 3). In uninjured fins, neutrophils can be observed in the vasculature of the fin (Fig. 3A,F). In the amputation model, no remarkable change in the distribution of *mpx:GFP*-positive cells was observed at different time points after resection (Fig. 3B-E). By contrast, at 10 min post-cryoinjury (mpci) and 6 hpci, no *mpx:GFP* expression was detected at the site of cryoinjury, indicating destruction of the blood cells by freezing/thawing (Fig. 3G,H). At 24 hpci, *mpx:GFP-*positive blood cells started to reappear in the injury zone (Fig. 3I′). Importantly, the proximal intact part of the fin accumulated large numbers of neutrophils, as compared to the stump after amputation at this time point (Fig. 3D″,I″). At 4 dpci, neutrophils invaded the margin of the truncated stump (Fig. 3J′). Moreover, they were markedly increased in the base of the fin in comparison to the amputation model, indicating an inflammatory response (Fig. 3E″,J″). Thus, cryoinjury triggers an enhanced inflammatory response in the remaining part of the fin as compared to the amputation model.

### Concomitant clearance of tissue debris and cellular proliferation in the stump define the reparative phase after cryoinjury

**Fig. 4.**
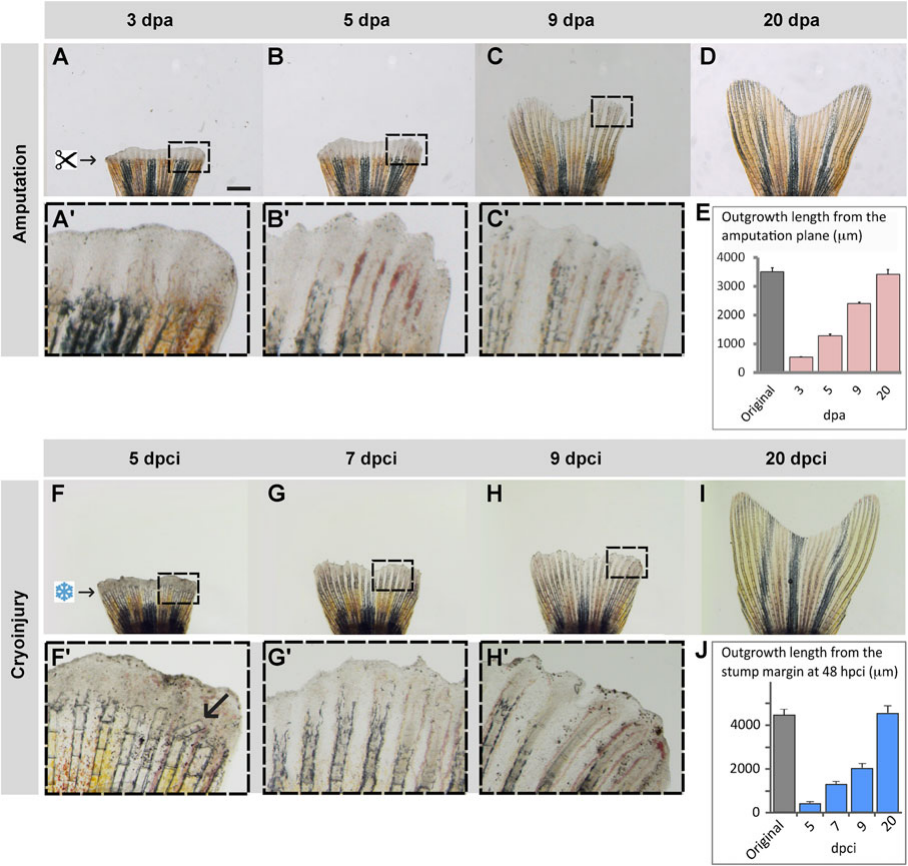
**Truncation of the damaged fin tissue is followed by resumed regeneration.** (A-D) Time-lapse imaging of fins after amputation during the outgrowth-formation, with boxed areas magnified in A′-C′. (F-I) Time-lapse imaging of fins after cryoinjury during the regenerative phase, with boxed areas magnified in F′-H′. Despite a delay of fin loss and partial damage of the stump, the regenerate reproduces a normal shape of the original fin within 20 days. (F′) Arrow indicates a broken bone. (E,J) Quantification of the fin regeneration after amputation (E) and cryoinjury (J). The length of the 3 longest lateral rays was measured from the stump margin after fin loss to the distal tip of the regenerate at different time-points. Error bars represent s.e.m., *N*=4 fins. Scale bar in A=1 mm.

To investigate the regenerative capacity after cryoinjury-induced damage, we continued the time-lapse imaging of the fin stumps after the initial 2 days (Fig. 1B,C). In the fin amputation model, the regenerative outgrowth becomes clearly visible already at 3 dpa (Fig. 4A,A′). It comprises a spatio-temporally organized field of cells with the developmental plasticity for reconstruction of the missing parts (Pfefferli and Jaźwińska, 2015; Wehner and Weidinger, 2015). We found that after cryoinjury, a blastemal outgrowth started to form above the remaining fin at 5 dpci (Fig. 4F). Magnified images revealed that the new tissue contained broken bones and dark necrotic-like patches of cells (Fig. 4F′). Despite these disturbances, the outgrowth progressed throughout regeneration and acquired an appearance comparable to that observed after amputation at day 9 (Fig. 4B,C,G,H). The regeneration was accomplished at 20 dpci, at the same time for amputated fins (Fig. 4D,E,I,J). Thus, extensive tissue death, blood clot deposition and inflammation during several days after cryoinjured did not prevent a normal subsequent progression throughout the regeneration.

**Fig. 5.**
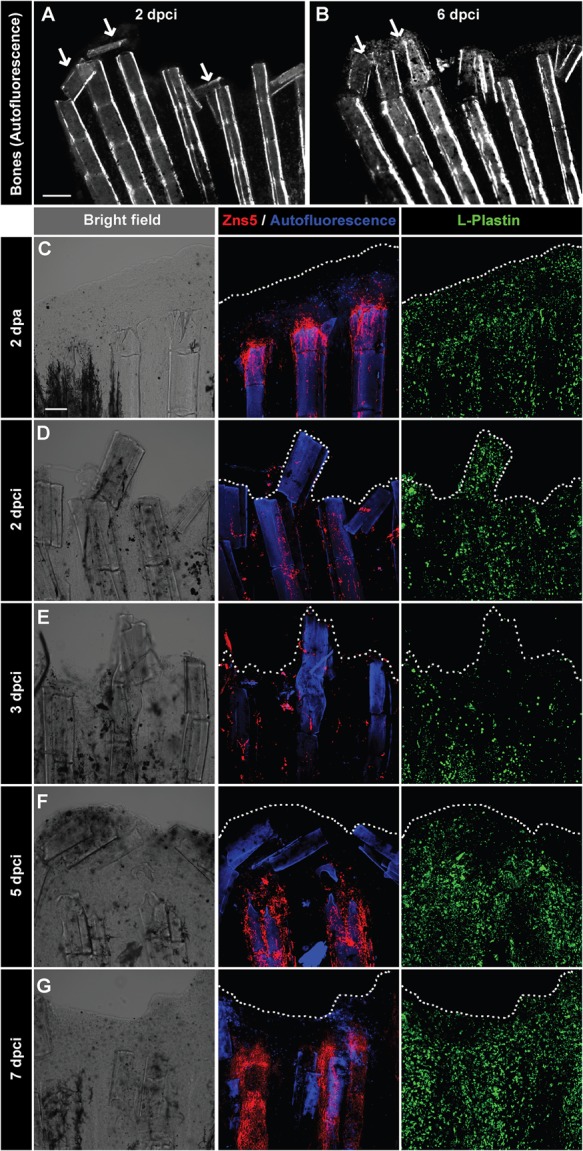
**Detachment of the destroyed fin tissue is associated with displacement and resorption of the dead bone fragments at the wound margin.** (A,B) Imaging of bones in the same fin detected by autofluorescence of the mineralized matrix at 2 and 6 dpci. The margin of the remnant fins contains detached and displaced bone fragments between the rays that become resolved (arrows). *N*=5. (C-G) Confocal imaging of whole-mount fins immunostained with the osteoblast marker Zns5 (red), phagocyte marker L-plastin (green) and autofluorescent bone matrix (blue) at 2 dpa (C) and at different time points after cryoinjury (D-G). At 2 dpa (C), Zns5-labelled osteoblasts accumulate at the tip of the bone to initiate bone regeneration. L-plastin-expressing cells are present in the entire tissue. At 2 dpci (D) and 3 dpci (E), osteoblasts are scattered along the bones in irregular manner. At 5 dpci (F), Zns5-positive cells are enriched at the tips of the intact bones, below the margin of the stump that contains bone debris devoid of osteoblasts. At 7 dpci (G), Zns5 immunostaining is robustly enhanced along the remaining bones, indicating resumed regeneration. L-plastin-expressing cells are associated with the repairing and regenerating tissue. *N*=4. Scale bar in A=200 µm, in C=100 µm.

To characterize the defects in cryoinjured fins, we analysed the morphology of the remaining bones after tissue sloughing. Live imaging of fins at 2 dpci revealed an accumulation of bone fragments at the margin of the stump (Fig. 5A). The tips of the bones were released from the rays and often displaced perpendicularly to the original position. Imaging of the same fins after the next 4 days (at 6 dpci) revealed that the remnants of mineralized matrix were undergoing degradation and resorption (Fig. 5B). To test whether the detached bone segments were associated with bone-producing cells, the osteoblasts, we performed immunofluorescence staining with Zns5 antibody and we labelled phagocytic cells with anti-L-plastin antibody of whole-mount fins combined with autofluorescence of bone matrix (Fig. 5C-G). In the stump of amputated fins at 2 dpa, Zns5 immunoreactivity was increased at the tips of the bones, indicating the dedifferenatiation of osteoblasts (Fig. 5C). By contrast, at days 2 and 3 after cryoinjury, such a population of Zns5-positive osteoblasts was not observed (Fig. 5D-E). Indeed, the visualization of mature osteoblasts in transgenic reporter fish (*osteocalcin:GFP*) revealed destruction of bone-producing cells along the cryoinjured plane (Fig. S2). Thus, after cryoinjury, the regenerating margin needs to cope with the presence of the displaced matrix remnants of the destroyed bone segments.

Despite of the massive bone damage, an accumulation of Zns5-positive cells was observed at 5 and 7 dpci (Fig. 5F,G). This finding demonstrates that the regeneration programme was resumed with a delay of 3 days in comparison to fins after amputation. To evaluate whether phagocytic cells were involved in the resorption of bone debris, we analysed L-plastin immunostaining. In both injury models, amputation and cryoinjury, phagocytes were detected in the stump and the regenerating tissue (Fig. 5C-G). However, an enhanced accumulation of L-plastin cells occurred at 5 and 7 dpci (Fig. 5F,G), indicating clearance of the debris during the transition to the regenerative outgrowth phase.

**Fig. 6.**
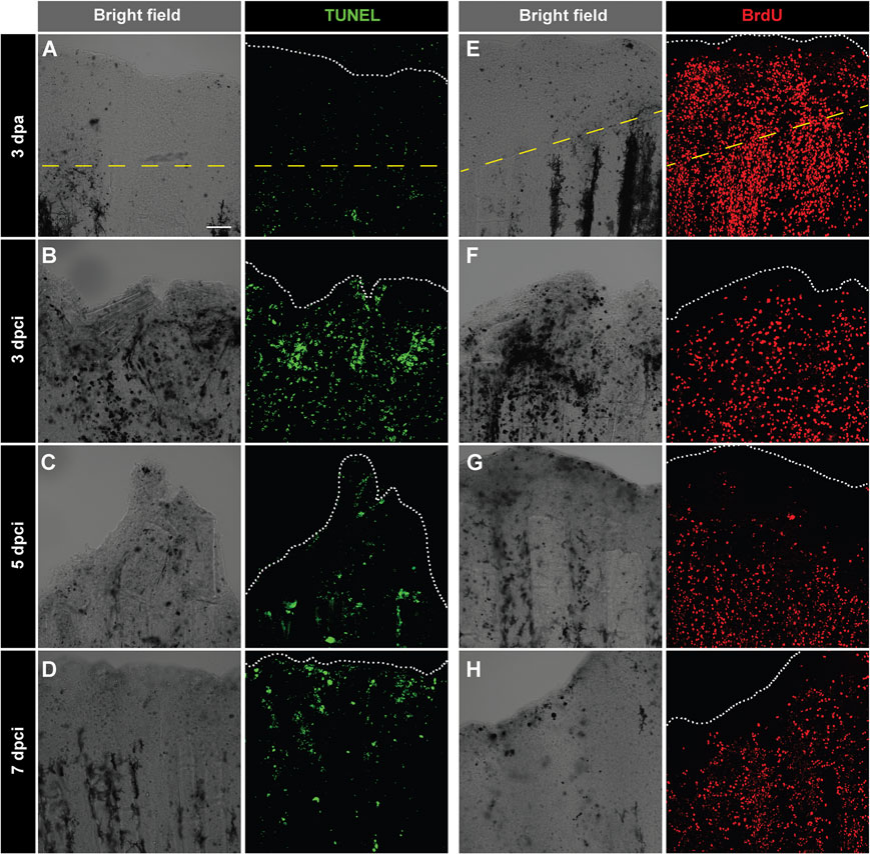
**Enhanced cell proliferation and upregulation of tissue remodelling protein during regeneration of cryoinjured fins.** (A-D) TUNEL labelling (green) of whole-mount fins at 3 dpa (A) and at different time points after cryoinjury (B-D). At 3 dpa (A), TUNEL staining is nearly absent. At 3 dpci (B), 5 dpci (C) and 7 dpci (D), tissue debris (dark structures in bright-field images) are associated with TUNEL-positive cells. (E-H) Immunodetection of BrdU (red) in whole-mount caudal fins at 3 dpa (E) and at different time points after cryoinjury (F-H). As compared to 3 dpa (E), BrdU-incorporation is lower in cryoinjured fins at 3 dpci (F), especially at the position of necrotic cells (dark regions in the bright-field). Cell proliferation becomes more abundant at 5 dpci (G) and 7 dpci (H). The dashed yellow line indicates the plane of amputation. The edge of the fin is indicated with a white dotted line. *N*=5. Scale bar=100 µm.

To determine whether the formation of the outgrowth is associated with apoptosis, we first performed a TUNEL assay on whole-mount fins. In fins after amputation, at 3 dpa, we observed only few scattered TUNEL-positive cells (Fig. 6A). However, after cryoininjury, at 3, 5 and 7 dpci, the margin of the regenerating stump displayed the remarkable presence of TUNEL-labelled cells (Fig. 6B-D). Thus, the transition from the reparative to the regenerative phase is associated with the continuous apoptotic elimination of cells in the partially damaged part of the stump.

Then, to investigate cell proliferation, we performed a BrdU proliferation assay. The analysis of truncated whole-mount fins at 3 dpci revealed markedly lower cell proliferation, as compared to the amputation model at 3 dpa (Fig. 6E,F). Interestingly, at 5 dpci, the partially damaged region contained fewer proliferating cells than the proximal non-damaged part (Fig. 6G). At 7 dpci, cell proliferation expanded towards the fin margin, reaching a similar pattern to the one after amputation (Fig. 6H).

**Fig. 7.**
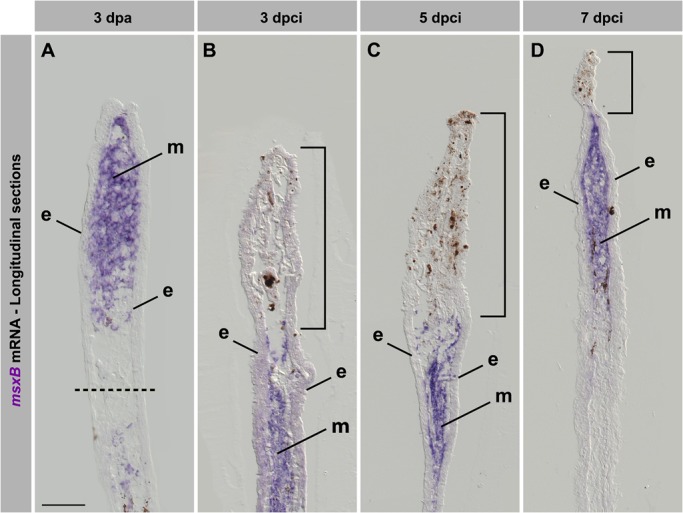
**The blastemal marker *msxB* is upregulated in regenerating fins after cryoinjury.** (A-D) *In situ* hybridization of longitudinal fin sections using *msxB* antisense probe (purple) at 3 dpa (A) and different time points after cryoinjury (B-D). Dashed line indicates the amputation plane. Bracket indicates the damaged tissue remaining in the cryoinjured stump. In the amputation model, *msxB* is upregulated in the mesenchyme of the outgrowth. After cryoinjury, *msxB* expression is induced below the damaged tissue. At 7 dpci (D), a normal blastema is formed, despite small amounts of distally remaining tissue debris. e, epidermis; m, mesenchyme. *N*=4. Scale bar=100 µm.

To determine whether cell proliferation was associated with upregulation of blastema genes, we analysed the expression of *msxB*, which is a well-established marker of the undifferentiated mesenchyme in the regenerative outgrowth in the fin amputation model (Fig. 7A) (Akimenko et al., 1995). We found that *msxb* was induced after cryoinjury in the proximal part of the stump below the partially damaged tissue at 3 and 5 dpci (Fig. 7B,C). These results suggest that the mesenchymal cells are activated to form the blastema, despite a massive demolition of the distal tissue. Interestingly, at 7 dpci, when the damaged area was nearly completely resolved, the *msxB* expression reproduced the normal pattern that resembled the blastema in the amputation model at 3 dpa (Fig. 7A,D). We concluded that after cryoinjury, cellular proliferation and blastema formation are resumed in a delayed fashion as compared to the amputation model.

### The regenerative outgrowth phase occurs after the completion of the reparative phase

**Fig. 8.**
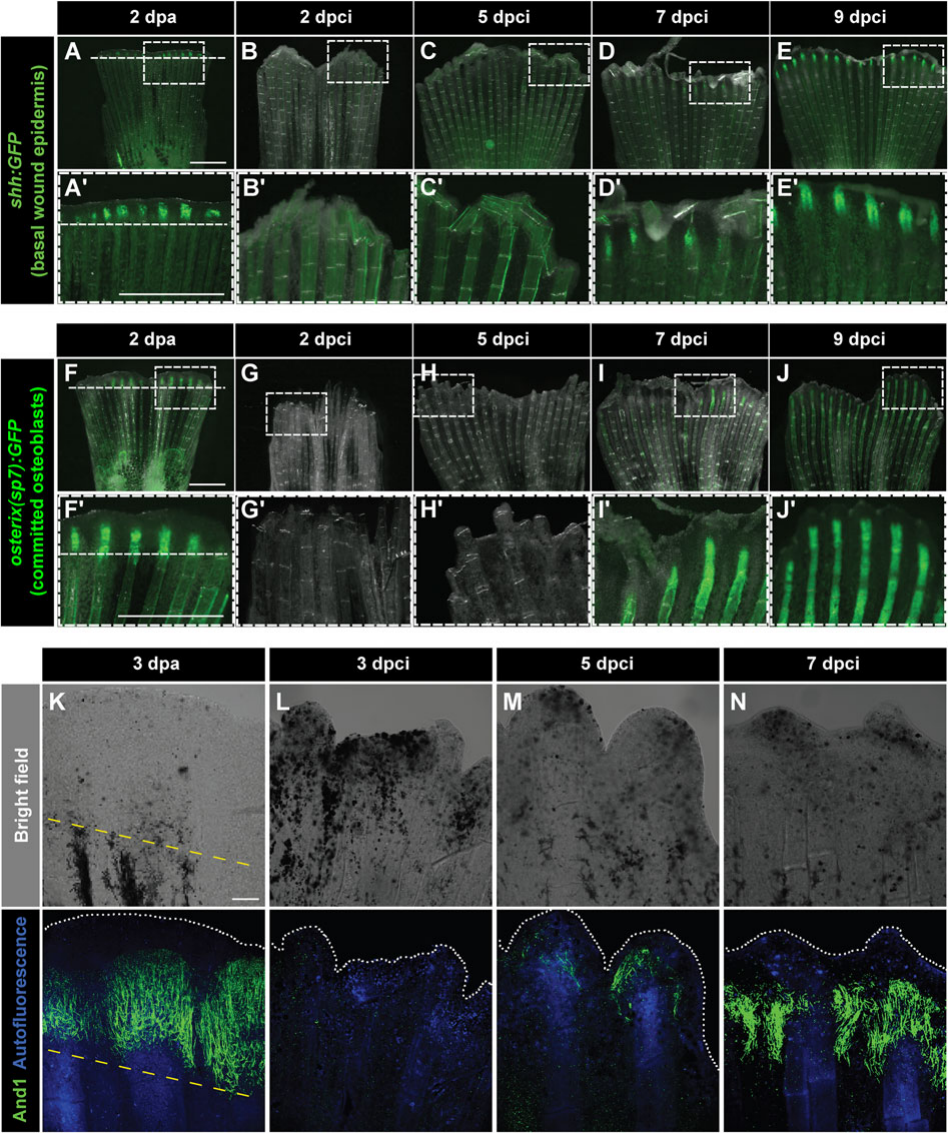
**Dynamics of bone and actinotrichia regeneration in cryoinjured fins.** (A-E′) Live-imaging of *shh:GFP* transgenic fish demarcates a subset of cells in the basal layer of the lateral wound epidermis (green) at 2 dpa (A) different time points after cryoinjury (B-E). Dashed line indicates the plane of amputation. The expression of *shh:GFP* becomes detectable starting at 7 dpci (D,D′), indicating organization and subdivision of the basal epithelium. *N*=4. Boxes in A-E magnified in A′-E′. (F-J′) Live-imaging of transgenic fish *osterix(sp7):GFP* highlights intermediately differentiated osteoblasts (green) at 2 dpa (F) and at different time points after cryoinjury (G-J). The expression of *osterix(sp7):GFP* becomes detectable starting at 7 dpci (I,I′), indicating bone regeneration. *N*=4. Boxes in F-J magnified in F′-J′. (K-N) Bright-field (upper panels) and confocal (lower panels) images of whole-mount fins immunostained with anti-And1 antibodies (green) at 3 dpa (K) and at different times after cryoinjury (L-N). Bone matrix is detected by autofluorescence (blue). The bright-field images show dark necrotic tissue at the margin of cryoinjured fins. The expression of And1 starts at 5 dpci (M) and becomes more evident at 7 dpci (N). *N*=4. Yellow dashed line indicates the amputation plane. Scale bar in A=1 mm and in K=100 µm.

The epithelial-mesenchymal interactions are fundamental to the execution of developmental and regenerative programmes after fin amputation (Blum and Begemann, 2012; Gemberling et al., 2013; Yoshinari and Kawakami, 2011). Sonic hedgehog (Shh) is one of the factors produced by the lateral basal wound epithelium of the outgrowth, suggested to be involved in regeneration of the underlying bones (Blum and Begemann, 2015; Laforest et al., 1998; Quint et al., 2002). To analyse the expression of *shh* after cryoinjury, we performed time-lapse imaging of *shh:GFP* transgenic fish. In the fin amputation model, *shh:GFP* has been shown to be induced at 2 dpa in the wound epithelium of the regenerating rays (Fig. 8A,A′) (Zhang et al., 2012). In contrast, the expression of the transgene was initiated only at 7 dpci in our injury method (Fig. 8B-D′). A robust expression of this transgenic reporter was observed at 9 dpci, indicating a substantial delay in the reactivation of the regenerative programme related to *shh* gene (Fig. 8E,E′). We concluded that the wound epithelial subdomains become organized after the completion of the reparative phase in the stump.

Next, we characterized the dynamics of bone regeneration using a reporter of committed immature osteoblasts of *osterix(sp7):GFP* transgenic fish. In the amputation model, the expression of *osterix(sp7):GFP* is clearly visible in newly forming bones of the outgrowth at 2 dpa (Fig. 8F,F′) (Knopf et al., 2011; Pfefferli et al., 2014). In the cryoinjury model, we detected the first signs of the *osterix(sp7):GFP* expression at 7 dpci, while a robust expression appeared at 9 dpci (Fig. 8G-J′). Thus, similarly to *shh:GFP,* the expression of *osterix(sp7):GFP* suggests a regenerative delay of approximately 4 days, as compared to the amputation model. We concluded that the patterning and regenerative morphogenesis take place after the completion of debris clearance.

Besides the bones, the skeleton of the zebrafish fin includes actinotrichia, which support the distal edge of the dermal fold (Durán et al., 2011; Zhang et al., 2010). To investigate the regeneration of these elastic spicules, we performed immunostaining with an anti-Actinodin 1 (And1) antibody (Fig. 8K-N). At 3 dpa, strong labelling of And1 was detected in extracellular actinotrichial fibers above the amputation plane (Fig. 8K). We found that after cryoinjury, actinotrichia formation was only initiated at 5 dpci (Fig. 8L,M). Importantly, the And1-positive structures were distributed at a more proximal position from the fin edge, probably due to the presence of damaged tissue at the tip of the stump. However, at 7 dpci, after resorption of bone debris, the expression of And1 progressively reached the distal-most margin of the fin, leading to the restoration of a normal pattern of actinotrichia (Fig. 8N). Taken together, both fin skeletal elements, bones and actinotrichia, start to regenerate after the reparative phase following cryoinjury.

## DISCUSSION

**Fig. 9.**
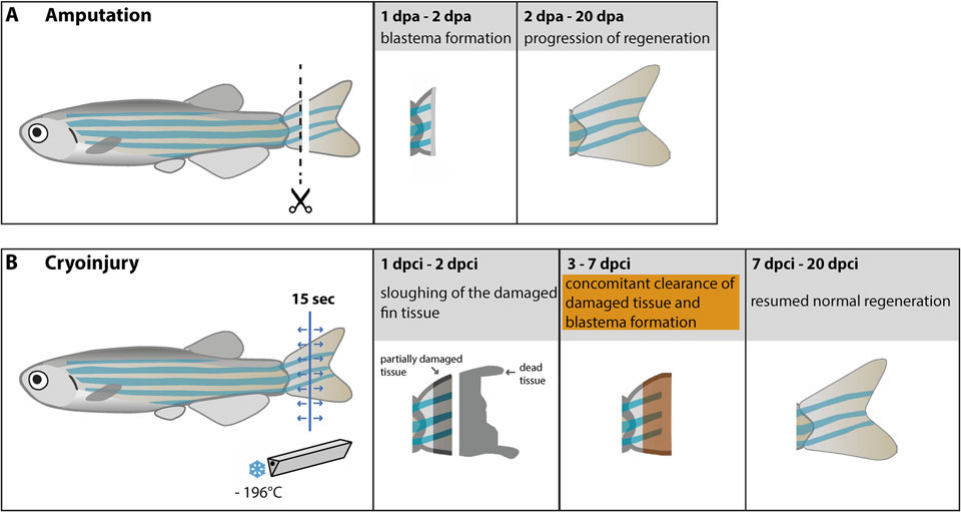
**Schematic comparison of the fin amputation and fin cryoinjury experimental models.** (A) The surgical removal of the fin by amputation results in rapid wound epithelium and blastema formation within 2 dpa, followed by complete fin regeneration in 20 days. (B) The cryoinjury model is based on destruction of the fin by touching with a cold blade for 15 s along the cryoinjury plane (blue line). Spreading of cold in the tissue is indicated by arrows. Within 1 to 2 dpci, the destroyed part of the tissue (unpigmented grey zone) undergoes natural sloughing. Below the truncation plane, the stump contains partially damaged tissue with broken bones and with extensive inflammation (shaded part of the fin). At 3 to 7 dpci, the reparative/regenerative phase takes place, whereby the dead material is degraded and the regenerative outgrowth becomes established (orange zone). After this period, regeneration is rapidly resumed and completed at 20 dpci.

The amputation procedure causes relatively little damage to the remaining stump of the zebrafish fin, allowing for rapid re-establishment of the epidermal barrier between the environment and the internal tissues. By contrast, wound healing in tetrapod appendages requires more time, and includes additional responses, namely blood clotting, inflammation and tissue demolition at the distal stump (Fernando et al., 2011; Godwin and Rosenthal, 2014; Monaghan and Maden, 2013; Simkin et al., 2015a). Importantly, these processes are observed both in the regenerative context of urodele limb and murine digits, as well as in non-regenerative repair by scarring. In this study, we investigated the impact of a prolonged wound healing response on the regenerative capacity of the zebrafish fin. We established a new cryoinjury model that triggers a spontaneous fin loss due to extensive tissue death, followed by blood clotting, osteolysis and inflammation. This model provides additional phases to the process of fin regeneration, which have not been observed in the amputation model. First, the dead material becomes spontaneously sloughed between 12 and 48 hpci. Second, the partially damaged tissue simultaneously undergoes clearance of debris and re-establishment of the regenerative programmes during the subsequent 3 to 7 days (Fig. 9). Despite the perturbations, the fin regeneration was normally completed after 20 dpci, at a similar time to that found during fin regeneration after amputation. This study demonstrates that massive tissue destruction markedly delays the initiation of regeneration via the addition of a reparative phase, which, however, does not disturb the timing of the subsequent regenerative phase (Fig. 8).

In our laboratory, we have previously established a cryoinjury model to study heart regeneration in zebrafish (Chablais and Jazwinska, 2012a; Chablais et al., 2011). The freezing procedure induces ice crystal formation, which disrupts the cellular integrity after thawing (Gao and Critser, 2000). As opposed to the resection model, this injury method does not rely on the immediate surgical removal of the tissue, but on the delayed organ loss and prolonged degradation of the dead tissue. In this situation, two distinct processes have to be coordinated, namely degradation of the dead material and generation of new tissue. In the case of the zebrafish heart, several laboratories have demonstrated a transient deposition of fibrotic tissue after cryoinjury (Chablais et al., 2011; Gonzalez-Rosa et al., 2011; Schnabel et al., 2011). Indeed, this provisional fibrosis is considered to play a beneficial mechanical role by increasing robustness of the injured myocardial wall, which has to continuously maintain the blood-pumping effort (Chablais and Jazwinska, 2012b). To date, no scarring has been reported after fin injury. Our analysis did not reveal collagenous fibrotic tissue deposition after fin cryolesion (data not shown). Thus, as opposed to the heart, the reparative phase of fin regeneration occurs in a scarless manner.

In addition to the freezing/thawing effect along the cryoinjury, we observed tissue loss at the distal margin of the fin, which was not directly affected by the cold. Our data indicated that the cause of this damage was dependent on the interruption of blood circulation at the cryoinjury plane. Thus, the distal region was probably damaged by ischemia, which led to cell apoptosis and natural truncation of the dead part of the appendage. Interestingly, the spontaneous sloughing of the dead tissue did not eliminate all of the damaged cells, resulting in a mixture of intact and apoptotic cells in the remaining stump. The partially damaged region of the stump displayed abnormal pigmentation, patches of dying cells and broken, misplaced bones. The TUNEL assay revealed elevated cell death in the partially damaged zone during the reparative phase. The histolytic aspect of this phase was particularly evident by the osteolysis of bone fragments between 3 and 7 dpci. Surprisingly, extensive tissue demolition and disorganization coincided with the enhanced proliferation of remaining healthy cells that succeeded in replacing the damaged cells. Thus, the zebrafish fin displays a remarkable capacity to simultaneously cope with the resorption of distorted tissue and formation of new structures.

In the cryoinjured fins, the regenerative programmes were re-established in a delayed manner compared to amputated fins, as visualized by the expression of the wound epithelial signal *shh:GFP*, the reactivation of developmental genes in osteoblasts, such as the *osterix:GFP* reporter, and regeneration of actinotrichia that contain Actinodin 1. The timing of the reactivation of these regeneration markers coincided with the termination of debris clearance at 7 dpci. This situation is reminiscent of the scenario of murine digit regeneration, during which the histolysis and new structure replacement occur in a non-overlapping sequential manner (Simkin et al., 2015a,b). Elucidation of the factors involved in the switch from the degradation phase to the regenerative phase is important for understanding vertebrate appendage regeneration.

The zebrafish fin has been shown to possess a very robust ability to restore its bones after crush injury (Sousa et al., 2012). This injury model is reminiscent of bone fracture repair in mammals. Similarly, our cryolesion model mimics several pathologic aspects of mammalian limb loss that disrupt the homeostasis of the stump. Understanding how tissue resorption and regeneration are synchronized in the zebrafish fin might provide new insights for regenerative biology and medicine.

## MATERIALS AND METHODS

### Animal procedures and fin cryoinjury

The present work was performed with fully grown adult fish at the age of 12-24 months. Adult zebrafish were maintained at 26.5°C in the water circulating system. Wild-type fish were AB (Oregon). The transgenic lines were Tg(*mpx*:*GFP*) (Loynes et al., 2010); *osterix(sp7):gfp* (*OlSp7:nlsGFP^zf132^*) (Spoorendonk et al., 2008); Tg(*osteocalcin:GFP*) (Knopf et al., 2011); Tg(*tie2:EGFP*) (Motoike et al., 2000) and Tg(*shh:GFP*) *2.4shh:gfpABC#15* (Zhang et al., 2012). For fin surgery, the animals were anesthetized in 0.6 mM tricaine (MS-222 ethyl-m-aminobenzoate, Sigma-Aldrich) and the caudal fins amputated using a razor blade or cryoinjured with a cryotome blade. The fin cryoinjury was performed with a steel cryotome blade, (22 cm, 260 g, Product 14021660077, Leica). The cryotome blade was immerged for 90 s in liquid nitrogen, removed and held for 5 s in the air to avoid the dispersion of liquid nitrogen droplets. The blade was applied by touching the fin surface for 15 s. For proliferation analysis, the fish were incubated for 7 h in fish water containing 50 µg/ml of BrdU (Sigma-Aldrich). The cantonal veterinary office of Fribourg has approved this experimental research on animals.

### Microscopy

Live imaging was performed with a stereomicroscope coupled to a Leica DCF425 C camera for colour images and a Leica DCF345 FX camera for fluorescence images. Imaging of immunostaining was performed with a Leica TCS SP5 confocal microscope.

### Terminal deoxynucleotidyl transferase dig-UTP nick end-labeling (TUNEL)

For TUNEL reactions on whole-mounts, samples were post-fixed for 10 min in 1% formalin, washed twice for 5 min in PBS and treated in precooled ethanol:acetic acid 2:1 for 5 min at −20°C. After two washes in PBS, fins were incubated in TdT reaction buffer (25 mM Tris-HCl, 200 mM sodium cacodylate, 0.25 mg/ml BSA, 1 mM cobalt chloride) for 10 min. DNA breaks were elongated with Terminal Transferase (Roche) and Digoxigenin-dUTP solution (Roche) during TdT reaction for 1 h at 37°C. The reaction was stopped by incubation in stop-wash buffer (300 mM NaCl, 30 mM sodium citrate) for 10 min, followed by two washes in PBS. The staining with anti-digoxigenin fluorescein conjugate was performed according to the manufacturer's protocol (Roche). After a wash in PBS, the samples were used for immunofluorescence as described below.

### Immunofluorescence

The immunofluorescence protocol was performed as described previously (Chablais and Jazwinska, 2010). The primary antibodies used were anti-BrdU at 1:100 (ab6326, Abcam, UK), anti-L-plastin at 1:2000 (a kind gift from P. Martin, University of Bristol, UK) (Kemp et al., 2010), Zns5 at 1:500 (ZIRC, USA), anti-Actinodin1 at 1:5000 (Thorimbert et al., 2015).

### In situ hybridization

To generate antisense probe, portions of the coding sequences of genes were cloned by the PCR amplification of zebrafish cDNA. The reverse primers were synthesized with an addition of the promoter for T3 polymerase. The following forward (F) and reverse (R) primers were used for *msxB* (NM_131260) F: gagaatgggacatggtcagg and R: gcggttcctcaggataataac (721 bp). Digoxigenin-labelled RNA antisense probes were synthesized from PCR products with the Dig labelling system (Roche). *In situ* hybridization on tissue cryosections was according to Sallin and Jaźwińska (2016).
